# ﻿Molecular phylogeny and morphology reveal two novel entomopathogenic species of Hypocreales (Polycephalomycetaceae and Cordycipitaceae), from China

**DOI:** 10.3897/mycokeys.127.176090

**Published:** 2026-01-16

**Authors:** Quan-Ying Dong, Nian-Kai Zeng, Jin-Na Zhou, Shun-Yu Gao, Cheng-Dong Xu, Zhen-Ji Wang

**Affiliations:** 1 College of Agronomy, Chuxiong Normal University, Chuxiong 675000, China Chuxiong Normal University Chuxiong China; 2 Ministry of Education Key Laboratory for Ecology of Tropical Islands, Key Laboratory of Tropical Animal and Plant Ecology of Hainan Province, College of Life Sciences, Hainan Normal University, Haikou 571158, China Hainan Normal University Haikou China

**Keywords:** Geographic distribution, new taxa, *
Pleurocordyceps
*, *
Samsoniella
*, taxonomy

## Abstract

Based on an integrated taxonomic approach combining multi-locus phylogenetic analysis and morphological characterization, we formally describe and illustrate two new entomopathogenic fungal species from China, *Pleurocordyceps longiphialis* and *Samsoniella aggestitenuipes*. Phylogenetic analyses of a six-locus dataset (ITS, nr SSU, nr LSU, *tef1-α*, *rpb1*, and *rpb2*) strongly support the distinct phylogenetic positions of the two new species within their respective genera. Morphologically, *P. longiphialis*, isolated from a Scarabaeoidea larva, possesses notably elongated α-phialides (9.5–101 µm) and dimorphic conidia. In addition, *S. aggestitenuipes*, obtained from a lepidopteran pupa, displays synnemata with powdery conidial masses and phialides measuring 6–28 µm. This study also reviews the host ranges and geographic distributions of *Pleurocordyceps* and *Samsoniella*. *Pleurocordyceps* species are known to infect insects in Coleoptera, Hemiptera, and Lepidoptera, and also parasitize fungi such as *Elaphomyces*, *Ophiocordyceps*, *Paraisaria*, and *Perennicordyceps*. *Samsoniella* species have been reported from Lepidoptera, Coleoptera, Hymenoptera, and Arachnida. Geographically, *Pleurocordyceps* occurs in Asia (China, Japan, and Thailand) and South America (Ecuador), while *Samsoniella* is distributed across East Asia (China, Laos, Thailand, and Vietnam), Europe (UK and Ireland), and South America (Argentina).

## ﻿Introduction

The taxonomic classification of entomopathogenic and mycoparasitic fungi has undergone significant revisions in recent years, driven by advances in molecular phylogenetics (Sung et al. 2007; Mongkolsamrit et al. 2020; Wang et al. 2020; Dong et al. 2022; Kuephadungphan et al. 2022; Tanaka et al. 2023; Tang et al. 2023; Xiao et al. 2023; Chuang et al. 2024). A significant milestone was the establishment of the family Polycephalomycetaceae by Xiao et al. (2023), which was identified as a phylogenetically distinct lineage sister to Ophiocordycipitaceae. This family includes the type genus *Polycephalomyces* and three other genera, *Dingleyomyces*, *Perennicordyceps*, and *Pleurocordyceps*, which together form a well-supported clade. Most recently, Wang et al. (2024b) described an additional genus, *Paradingleyomyces*, parasitizing stromata of Ophiocordyceps cf. cochlidiicola in Yunnan Province, China, further expanding the taxonomic breadth of the family. Taxa within Polycephalomycetaceae display notable ecological versatility, infecting a broad spectrum of hosts that include both insects and fungi (Mongkolsamrit et al. 2019; Xiao et al. 2023; Liu et al. 2024b).

Among these genera, *Pleurocordyceps* was established by Wang et al. (2021) based on integrative morphological and phylogenetic evidence. The type species, *Pleurocordyceps sinensis*, was originally described as *Paecilomyces sinensis* by Chen et al. (1984). Xiao et al. (2023) confirmed the delineation of its generic boundaries and familial placement, recognizing 22 accepted species (Index Fungorum, accessed December 2025; https://www.indexfungorum.org/Names/Names.asp). *Pleurocordyceps* species are obligate parasites of insects or fungi. The sexual morph is characterized by fleshy, stipitate stromata ranging from reddish-brown to yellow, bearing capitate fertile heads with immersed, pyriform to ovoid perithecia. Asci are cylindrical, thick-walled, and feature a distinct apical cap, producing filiform ascospores that disarticulate into uniform cylindrical secondary spores (Wang et al. 2021; Xiao et al. 2023). The asexual morph is hyphomycetous and highly polymorphic, occasionally forming synnemata with conidial masses. Two types of phialides (α and β) produce globose to ellipsoidal α-conidia aggregated in slimy heads, and fusiform β-conidia occurring singly or in chains (Matočec et al. 2014; Wang et al. 2021; Xiao et al. 2023).

A major taxonomic revision by Wang et al. (2021) addressed persistent nomenclatural inconsistencies, such as the misapplication of the name *Polycephalomyces formosus* due to the erroneous strain ARSEF 1424. Phylogenetic analyses supported the transfer of ten species to *Pleurocordyceps*: *P. agarica*, *P. aurantiacus*, *P. lianzhouensis*, *P. marginaliradians*, *P. nipponica*, *P. onorei*, *P. phaothaiensis*, *P. ramosopulvinatus*, *P. sinensis*, and *P. yunnanensis* (Wang et al. 2021). Although *Polycephalomyces ramosus* represents the earliest described name within the clade and groups phylogenetically with *Po. tomentosus* and *P. sinensis*, the latter was designated as the type species due to the more comprehensive morphological and ecological data available (Wang et al. 2021).

Within Cordycipitaceae, the genus *Samsoniella* was recently erected to accommodate a phylogenetically distinct clade of entomopathogenic fungi, named in honor of Professor Robert A. Samson for his significant contributions to mycological research (Mongkolsamrit et al. 2018). This genus is distinguished by its vivid red-orange stromata in the sexual morph and similarly pigmented, irregularly branched synnemata, and an Isaria-like asexual morph, with oval to fusiform conidia serving as a key diagnostic characteristic (Mongkolsamrit et al. 2018; Dong 2023). Recent taxonomic studies, particularly in China, have rapidly expanded the genus, which currently comprises 56 species, 38 of which have been described in the last 3 years (Index Fungorum, accessed 15 December 2025; https://www.indexfungorum.org/Names/Names.asp). The recognition and circumscription of this genus not only refine the taxonomy of insect-associated fungi but also underscore the continuing expansion of fungal diversity, with *S. inthanonensis* designated as the type species. Despite these advances, a synthetic overview integrating host range, morphological variation, and geographic distribution for these rapidly diversifying genera is still lacking, and numerous recently collected specimens await study.

## ﻿Materials and methods

### ﻿Specimen collection and fungal isolation

Fungal specimens were obtained from Guizhou and Sichuan Provinces, China. Each specimen was photographed, documenting the collection site, GPS coordinates, and altitude. Following surface debris removal, samples were placed in sterile containers and transported under refrigeration to the laboratory. Specimens were subsequently cleaned, assigned accession numbers, and air-dried before processing. *Pleurocordyceps* was isolated using two methods: (1) tissue isolation, involving surface sterilization of internal sclerotia with 75% ethanol, aseptic dissection into 2–3 mm segments, and transfer to PDA plates; and (2) ascospore isolation, in which stromata were sequentially sterilized with 75% ethanol and 30% H_2_O_2_, rinsed five times with sterile water, dried, and plated as 2–3 mm fragments. The PDA medium (200 g/L potato extract, 20 g/L dextrose, 20 g/L agar) was supplemented with streptomycin (0.1 g/L) and tetracycline (0.05 g/L) after autoclaving to inhibit bacterial growth. For *Samsoniella*, conidia were directly collected from wild synnemata and inoculated onto PDA. All cultures were incubated at 25 °C, and purified fungal isolates were maintained at 25 °C or stored on PDA slants at 4 °C for preservation.

Voucher specimens (accession nos. CXAC 0015–0020) and corresponding axenic cultures (accession nos. CXCC 0015–0020) are deposited at the College of Agronomy Herbarium (**CXAC**) and the Chuxiong Fungal Culture Collection (**CXCC**) at Chuxiong Normal University, China, and are available for taxonomic identification, molecular analysis, and future studies.

### ﻿Morphological characterization

Ecological data, including host/substrate associations and geographical origin, were documented. Fungal isolates from agar slants were inoculated onto potato dextrose agar (PDA) plates and incubated at 25 °C for several days. Colony morphology was assessed with emphasis on conidial arrangement, phialide structure, and pigment production. For microscopic examination, mycelial fragments from 14-day-old cultures were placed on 5 mm agar blocks, mounted on glass slides, and incubated in moist chambers to promote sporulation. Morphological characteristics of asexual structures, including conidiophores, phialides, and conidia, were observed and measured using an Olympus BX53 compound microscope (Olympus Corporation, Tokyo, Japan).

### ﻿Extraction of DNA, polymerase chain reaction (PCR), and molecular sequencing

Genomic DNA was extracted from fungal mycelia using a commercial plant DNA isolation kit (FORE GENE, China) and amplified via polymerase chain reaction (PCR) for six genetic regions: ITS, nr SSU, nr LSU, *tef1-α*, *rpb1*, and *rpb2*, with primers listed in Table 1. The PCR was performed in a 25 µL reaction mixture containing 2.5 µL of 10× PCR Buffer (2 mM Mg^2+^; Transgen Biotech), 0.25 µL of Taq DNA Polymerase (Transgen Biotech), 2 µL of dNTPs (2.5 mM), 1 µL of DNA template (~500 ng/μL), 1 µL of each primer (10 μM), and 17.25 µL of deionized water. Amplification was conducted in a BIO-RAD T100™ Thermal Cycler under the thermal cycling conditions described by Dong et al. (2025). The PCR products were electrophoretically verified, purified, and subjected to bidirectional Sanger sequencing at the Beijing Genomics Institute (Shenzhen, China) using the original amplification primers.

**Table 1.** PCR primers used in this study.

Gene	Primer (forward/reverse)	5’-Sequence-3’	References
nr SSU	NS1	GTAGTCATATGCTTGTCTC	White et al. 1990
	NS4	CTTCCGTCAATTCCTTTAAG	
nr LSU	LR5	ATCCTGAGGGAAACTTC	Vilgalys and Hester 1990; Rehner and Samuels 1994
	LR0R	GTACCCGCTGAACTTAAGC	
ITS	ITS5	GGAAGTAAAAGTCGTAACAAGG	White et al. 1990
	ITS4	TCCTCCGCTTATTGATATGC	
*tef-1α*	EF1α-EF	GCTCCYGGHCAYCGTGAYTTYAT	Bischoff et al. 2006; Sung et al. 2007
	EF1α-ER	ATGACACCRACRGCRACRGTYTG	
*rpb1*	RPB1-5’F	CAYCCWGGYTTYATCAAGAA	Bischoff et al. 2006; Sung et al. 2007
	RPB1-5’R	CCNGCDATNTCRTTRTCCATRTA	
*rpb2*	RPB2-5′F	CCCATRGCTTGTYYRCCCAT	Bischoff et al. 2006; Sung et al. 2007
	RPB2-5′R	GAYGAYMGWGATCAYTTYGG	

### ﻿Phylogenetic analyses

A six-locus dataset comprising the internal transcribed spacer (ITS), nuclear small and large ribosomal subunits (nr SSU and nr LSU), translation elongation factor 1-α (tef1-α), and the largest and second-largest subunits of RNA polymerase II (*rpb1* and *rpb2*) was assembled for phylogenetic reconstruction. Newly generated sequences were verified through BLAST searches against the GenBank database and combined with reference sequences from related taxa obtained from GenBank (Table 2). Each gene region was independently aligned using MAFFT v7.526 with the L-INS-i strategy (http://mafft.cbrc.jp/alignment/server/, accessed 1 August 2025), followed by manual refinement in BioEdit v7.7.1. The individual alignments were concatenated into a supermatrix using FASconCAT-G v1.06 (Kück and Longo 2014). Phylogenetic conflict among loci was assessed using the partition homogeneity test in PAUP v5.0 with 1000 replicates (p > 0.01). The optimal partitioning scheme and corresponding nucleotide substitution models were selected under the Bayesian Information Criterion (BIC) using PartitionFinder2 v2.0.0 with the greedy algorithm (Lanfear et al. 2016). Maximum likelihood (ML) analysis was performed with IQ-TREE v3.0.1 under the optimal partition scheme with the GTR+G+I model, and branch support was evaluated using 1000 ultrafast bootstrap replicates (Nguyen et al. 2015). Bayesian inference (BI) was conducted in MrBayes v3.2.7. The best-fit substitution model (GTR+G+I) was selected with MrModeltest v2.2 (Nylander 2004). Markov chain Monte Carlo (MCMC) simulations were performed for 5,000,000 generations, sampling every 1,000 generations (Ronquist and Huelsenbeck 2003). Convergence was assessed in Tracer v1.7.2 to ensure effective sample sizes (ESS) exceeded 200, with the first 25% of trees discarded as burn-in (Rambaut et al. 2018). The resulting phylogenetic trees were visualized and annotated in FigTree v1.4.4 (https://tree.bio.ed.ac.uk/software/figtree/), with final layout and editing performed in Adobe Illustrator CS6 following the graphical standards of Xie et al. (2023).

**Table 2.** Specimen information and GenBank accession numbers for sequences used in this study.

Current name	Voucher	GenBank accession number						References
		ITS	nr SSU	nr LSU	*tef-1α*	*rpb1*	*rpb2*	
*P. agarica*	YHH PA1305^T^	KP276651	KP276655	–	KP276659	KP276663	KP276667	Wang et al. 2015a
*P. agarica*	YHC PA1307	KP276654	KP276658	–	KP276662	KP276666	KP276670	Wang et al. 2015a
*P. aurantiacus*	MFLUCC 17-2113^T^	MG136916	MG136904	MG136910	MG136875	MG136866	MG136870	Xiao et al. 2018
*P. aurantiacus*	MFLU 17-1393^T^	MG136919	MG136907	MG136913	MG136877	MG136868	MG136873	Xiao et al. 2018
*P. clavisynnema*	GZLG 23-102^T^	OQ968788	–	OQ968796	OQ982009	–	–	Xiao et al. 2024
*P. clavisynnema*	GZCC 22-2042	OQ968789	OQ968805	OQ968797	OQ982008	OQ981998	OQ982004	Xiao et al. 2024
*P. kanzashianus*		AB027371	AB027325	AB027371	–	–	–	Nikoh and Fukatsu 2000
*P. lanceolatus*	GACP 17-2004^T^	OQ172076	OQ172110	OQ172046	OQ459726	OQ459754	OQ459800	Xiao et al. 2023
*P. lianzhouensis*	MFLU 17-1582^T^	MG136920	MG136908	MG136914	MG136878	MG136869	MG271931	Xiao et al. 2018
*P. litangensis*	YFCC 06109293^T^	PP410597	PP541902	PP410593	PP550103	PP697751	PP550107	Liu et al. 2024b
*P. litangensis*	YFCC 06109295	PP410600	PP541905	PP410596	PP550104	PP697754	PP550108	Liu et al. 2024b
***P. longiphialis***	**CXCC 0017^T^**	**PX517735**	**PX699297**	**PX517741**	**PX622199**	–	**PX622205**	**This study**
***P. longiphialis***	**CXCC 0018**	**PX517736**	**PX699298**	**PX517742**	**PX622200**	–	**PX622206**	**This study**
***P. longiphialis***	**CXCC 0019**	**PX517737**	**PX699299**	**PX517743**	**PX622201**	–	**PX622207**	**This study**
***P. longiphialis***	**CXCC 0020**	**PX517738**	**PX699300**	**PX517744**	**PX622202**	–	**PX622208**	**This study**
*P. marginaliradians*	MFLU 17-2276^T^	MG136921	MG136909	MG136915	MG136879	–	MG271930	Xiao et al. 2018
*P. multisynnema*	GZLG 23-101	OQ968792	OQ968802	OQ968800	–	–	OQ982002	Xiao et al. 2024
*P. multisynnema*	GZCC 22-2041	OQ968793	OQ968803	OQ968801	OQ982010	OQ981997	OQ982003	Xiao et al. 2024
*P. neoagarica*	GZLG 23-103	OQ968790	–	OQ968795	–	–	–	Xiao et al. 2024
*P. neoagarica*	GZCC 22-2043	OQ968791	OQ968804	OQ968794	OQ982007	OQ981996	OQ981999	Xiao et al. 2024
*P. nipponicus*	BCC 18108	KF049657	MF416624	MF416569	MF416517	MF416676	MF416462	Kepler et al. 2013
*P. nipponicus*	NBRC 101406	JN943301	JN941753	JN941388	–	JN992487	–	Schoch et al. 2012
*P. nutansis*	GACP 19-3019^T^	OQ172086	OQ172120	OQ172058	OQ459740	OQ459766	OQ459812	Xiao et al. 2023
*P. nutansis*	MFLU 21-0275^T^	OQ172073	OQ172119	OQ172048	OQ459739	OQ459765	OQ459811	Xiao et al. 2023
*P. onorei*	BRA CR23902^T^	KU898841	–	–	–	–	–	Crous et al. 2017b
*P. onorei*	BRA CR23904	KU898843	–	–	–	–	–	Crous et al. 2017b
*P. parvicapitata*	MFLU 21-0270	OQ172082	OQ172105	OQ172054	OQ459722	OQ459751	OQ459796	Xiao et al. 2023
*P. parvicapitata*	MFLU 21-0271^T^	OQ172083	OQ172106	OQ172055	OQ459723	OQ459752	OQ459797	Xiao et al. 2023
*P. phaothaiensis*	BCC 84553^T^	MF959733	–	MF959737	MF959742	MF959745	–	Crous et al. 2017a
*P. phaothaiensis*	BCC 84552	MF959732	–	MF959736	MF959740	MF959744	–	Crous et al. 2017a
*P. ramosopulvinatus*	EFCC 5566	KF049658	–	KF049627	KF049682	KF049645	–	Kepler et al. 2013
*P. ramosopulvinatus*	SU 65	–	–	DQ118742	DQ118753	DQ127244	–	Chaverri et al. 2005
*P. sanduensis*	GZLG 23-104^T^	OQ968786	–	OQ968798	OQ982005	–	OQ982000	Xiao et al. 2024
*P. sanduensis*	GZCC 22-2044	OQ968787	OQ968806	OQ968799	OQ982006	OQ981995	OQ982001	Xiao et al. 2024
*P. sinensis*	ARSEF 1424	KF049661	KF049615	KF049634	KF049689	KF049651	KF049671	Kepler et al. 2013
*P. sinensis*	CN 80-2^T^	HQ832884	HQ832887	HQ832886	HQ832890	HQ832888	HQ832889	Wang et al. 2012
*P. sinensis*	GACP 20-2305	OQ172075	OQ172108	OQ172045	OQ459725	OQ459753	OQ459799	Xiao et al. 2023
*Pleurocordyceps* sp.	BCC 2637	KF049663	KF049619	KF049637	KF049693	–	KF049675	Kepler et al. 2013
*Pleurocordyceps* sp.	JB07.08.16_08	KF049662	KF049616	KF049635	KF049690	KF049652	KF049672	Kepler et al. 2013
*Pleurocordyceps* sp.	JB07.08.17_07b	–	KF049617		KF049691	KF049653	KF049673	Kepler et al. 2013
*Pleurocordyceps* sp.	NBRC 109987	AB925947	–	AB925983	–	–	–	Unpublished
*Pleurocordyceps* sp.	NBRC 109988	AB925948	–	AB925984	–	–	–	Unpublished
*Pleurocordyceps* sp.	NBRC 109990	AB925929	–	AB925968	–	–	–	Unpublished
*Pleurocordyceps* sp.	NBRC 110224	AB925931	–	AB925969	–	–	–	Unpublished
*Pleurocordyceps* sp.	GIMCC 3.570	–	JX006097	JX006098	JX006100	JX006101	–	Unpublished
*Pleurocordyceps* sp.	–	HM135164	HM135166	HM135165	–	–	–	Unpublished
*P. yunnanensis*	YHCPY1005	KF977848	KF977848	KF977848	KF977850	KF977852	KF977854	Wang et al. 2015b
*P. yunnanensis*	YHHPY1006^T^	KF977849	KF977849	KF977849	KF977851	KF977853	KF977855	Wang et al. 2015b
***S. aggestitenuipes***	**CXCC 0015^T^**	**PX517733**	**PX696762**	**PX517739**	**PX622197**	**PX622209**	**PX622203**	**This study**
***S. aggestitenuipes***	**CXCC 0016**	**PX517734**	**PX696763**	**PX517740**	**PX622198**	**PX622210**	**PX622204**	**This study**
*S. alboaurantium*	CBS 262.58^T^	AY624179	–	MG665232	JQ425685	–	–	Mongkolsamrit et al. 2018
*S. alboaurantium*	CBS 240.32	AY624178	–	JF415979	JF416019	JN049895	JF415999	Mongkolsamrit et al. 2018
*S. alpina*	YFCC 5818^T^	–	MN576753	MN576809	MN576979	MN576869	MN576923	Wang et al. 2020
*S. alpina*	YFCC 5831	–	MN576754	MN576810	MN576980	MN576870	MN576924	Wang et al. 2020
*S. anhuiensis*	RCEF2830^T^	–	OM268843	OM268848	OM483864	OM751889	–	Wang et al. 2024a
*S. anhuiensis*	RCEF2590	–	OR978313	OR978316	OR966516	OR989964	–	Wang et al. 2024a
*S. antleroides*	YFCC 6113	–	MN576748	MN576804	MN576974	MN576864	MN576918	Wang et al. 2020
*S. antleroides*	YFCC 6016^T^	–	MN576747	MN576803	MN576973	MN576863	MN576917	Wang et al. 2020
*S. aranea*	RCEF2831	–	OM268844	OM268849	OM483865	OM751882	OM802500	Wang et al. 2024a
*S. aranea*	RCEF2868	–	OM268845	OM268850	OM483866	OM751883	OM802501	Wang et al. 2024a
*S. araneicola*	DY05471^T^	PV082759	–	PV082876	PV171277	–	PV171197	Chen et al. 2025
*S. araneicola*	DY05472	PV082760	–	PV082877	PV171278	–	PV171198	Chen et al. 2025
*S. asiatica*	YFCC 869^T^	OQ476473	–	–	OQ506153	OQ506195	OQ506187	Wang et al. 2023
*S. asiatica*	YFCC 870	OQ476474	–	–	OQ506154	OQ506196	OQ506188	Wang et al. 2023
*S. aurantia*	TBRC 7271^T^	MF140764	–	MF140728	MF140846	MF140791	MF140818	Mongkolsamrit et al. 2018
*S. aurantia*	TBRC 7272	MF140763	–	MF140727	MF140845	–	MF140817	Mongkolsamrit et al. 2018
*S. cardinalis*	YFCC 5830	–	MN576732	MN576788	MN576958	MN576848	MN576902	Wang et al. 2020
*S. cardinalis*	YFCC 6144^T^	–	MN576730	MN576786	MN576956	MN576846	MN576900	Wang et al. 2020
*S. coccinellidicola*	YFCC 8772^T^	–	ON563166	ON621670	ON676514	ON676502	ON568685	Wang et al. 2022
*S. coccinellidicola*	YFCC 8773	–	ON563167	ON621671	ON676515	ON676503	ON568686	Wang et al. 2022
*S. coleopterorum*	A19501^T^	MT626376	–	–	MN101586	MT642600	MN101585	Chen et al. 2020
*S. cristata*	YFCC 6023	OQ476480	MN576736	MN576792	MN576962	MN576852	MN576906	Wang et al. 2020
*S. cristata*	YFCC 7004^T^	OQ476481	MN576737	MN576793	MN576963	MN576853	MN576907	Wang et al. 2020
*S. duyunensis*	DY07501	OR263188	–	OR263307	OR282780	OR282773	OR282776	Chen et al. 2023
*S. duyunensis*	DY07502	OR263189	–	OR263427	OR282781	–	OR282777	Chen et al. 2023
*S. erucae*	KY 11121^T^	ON502828	–	ON502835	ON525425	–	ON525424	Chen et al. 2022
*S. erucae*	KY 11122	ON502847	–	ON502822	ON525427	–	ON525426	Chen et al. 2022
*S. fanjingensis*	TR05241^T^	PV082761	–	PV082878	PV171279	–	PV171199	Chen et al. 2025
*S. fanjingensis*	TR05242	PV082762	–	PV082879	PV171280	–	PV171200	Chen et al. 2025
*S. farinospora*	YFCC 8774^T^	–	ON563168	ON621672	ON676516	ON676504	ON568687	Wang et al. 2022
*S. farinospora*	YFCC 9051	–	ON563169	ON621673	ON676517	ON676505	ON568688	Wang et al. 2022
*S. fusiformispora*	RCEF5406	–	OM268846	OM268851	–	OM751890	–	Wang et al. 2024a
*S. fusiformispora*	RCEF2588^T^	–	OR978312	OR978315	–	–	–	Wang et al. 2024a
*S. guiyangensis*	KY45341^T^	PV082763	–	PV082880	PV171281	–	PV171201	Chen et al. 2025
*S. guiyangensis*	KY45342	PV082764	–	PV082881	PV171282	–	PV171202	Chen et al. 2025
*S. guizhouensis*	KY 11161^T^	ON502823	–	ON502830	ON525429	–	ON525428	Chen et al. 2022
*S. guizhouensis*	KY 11162	ON502845	–	ON502846	ON525431	–	ON525430	Chen et al. 2022
*S. haniana*	YFCC 8769^T^	–	ON563170	ON621674	ON676518	ON676506	ON568689	Wang et al. 2022
*S. haniana*	YFCC 8770	–	ON563171	ON621675	ON676519	ON676507	ON568690	Wang et al. 2022
*S. houerensis*	KY45141^T^	PV082765	–	PV082882	PV171283	–	PV171203	Chen et al. 2025
*S. houerensis*	KY45142	PV082766	–	PV082883	PV171284	–	PV171204	Chen et al. 2025
*S. hymenopterorum*	A19521	MN128224	–	–	MN101588	MT642603	–	Chen et al. 2020
*S. hymenopterorum*	A19522^T^	MN128081	–	–	MN101591	MN101589	–	Chen et al. 2020
*S. inthanonensis*	TBRC 7915	MF140761	–	MF140725	MF140849	MF140790	MF140815	Mongkolsamrit et al. 2018
*S. jiangkouensis*	TR05031^T^	PV082767	–	PV082884	PV171285	PV171149	PV171205	Chen et al. 2025
*S. jiangkouensis*	TR05032	PV082768	–	PV082885	PV171286	PV171150	PV171206	Chen et al. 2025
*S. kaiyangensis*	KY45381^T^	PV082769	–	PV082886	PV171287	PV171151	PV171207	Chen et al. 2025
*S. kaiyangensis*	KY45382	PV082770	–	PV082887	PV171288	PV171152	PV171208	Chen et al. 2025
*S. kunmingensis*	YHH 16002^T^	–	MN576746	MN576802	MN576972	MN576862	MN576916	Wang et al. 2020
*S. lanmaoa*	YFCC 6193	–	MN576734	MN576790	MN576960	MN576850	MN576904	Wang et al. 2020
*S. lanmaoa*	YFCC 6148^T^	–	MN576733	MN576789	MN576959	MN576849	MN576903	Wang et al. 2020
*S. lasiocampidarum*	NTUPPMCC 20-062^T^	MT974208	–	MT974361	MW200218	MW200227	MW200236	Chuang et al. 2024
*S. lasiocampidarum*	NTUPPMCC 20-063	MT974210	–	MT974363	MW200219	–	MW200238	Chuang et al. 2024
*S. lepidopterorum*	DL 10071^T^	MN128076	–	–	–	MN101592	–	Chen et al. 2020
*S. lepidopterorum*	DL 10072	MN128084	–	–	–	–	–	Chen et al. 2020
*S. lurida*	HKAS144387^T^	PQ492700	PQ492707	PQ492339	PQ499065		PQ499078	Bu et al. 2025
*S. lurida*	HKAS144388	PQ492701	PQ492708	PQ492340	PQ499066	PQ499072	PQ499079	Bu et al. 2025
*S. miaolingensis*	DY05811^T^	PV082771	–	PV082888	PV171289	PV171153	PV171209	Chen et al. 2025
*S. miaolingensis*	DY05812	PV082772	–	PV082889	PV171290	PV171154	PV171210	Chen et al. 2025
*S. neopupicola*	KY 11322	ON502834	–	ON502833	ON525435	–	ON525434	Chen et al. 2022
*S. neopupicola*	KY 11321^T^	ON502843	–	ON502839	ON525433	–	ON525432	Chen et al. 2022
*S. pseudogunnii*	GY 407202	MZ831863	–	MZ831865	MZ855234	–	MZ855240	Chen et al. 2021
*S. pseudogunnii*	GY 407201	MZ827470	–	MZ827010	MZ855233	–	MZ855239	Chen et al. 2021
*S. pseudotortricidae*	YFCC 9052^T^	–	ON563173	ON621677	ON676521	ON676509	ON568692	Wang et al. 2022
*S. pseudotortricidae*	YFCC 9053	–	ON563174	ON621678	ON676522	ON676510	ON568693	Wang et al. 2022
*S. pupicola*	DY 101682	MZ827008	–	MZ827635	MZ855232	–	MZ855238	Chen et al. 2021
*S. pupicola*	DY 101681	MZ827085	–	MZ827009	MZ855231	–	MZ855237	Chen et al. 2021
*S. ramosa*	YFCC 6020^T^	–	MN576749	MN576805	MN576975	MN576865	MN576919	Wang et al. 2020
*S. sanmingense*	CGMCC3.25661	–	PP177395	PP179392	PP482033	PP464664	PP464647	Pu et al. 2025
*S. sanmingense*	CGMCC3.25662 ^T^	–	PP177396	PP179393	PP482034	PP464665	PP464648	Pu et al. 2025
*S. sapaensis*	YFCC 873^T^	OQ476489	–	–	OQ506152	OQ506194	OQ506186	Wang et al. 2023
*S. sapaensis*	YFCC 872	OQ476488	–	–	OQ506151	OQ506193	OQ506185	Wang et al. 2023
*S. simplicola*	DY45121^T^	PV082773	–	PV082890	PV171291	PV171155	–	Chen et al. 2025
*S. simplicola*	DY45122	PV082774	–	PV082891	PV171292	PV171156	–	Chen et al. 2025
*S. sinensis*	YFCC 8766^T^	–	ON563175	ON621679	ON676523	ON676511	ON568694	Wang et al. 2022
*S. sinensis*	YFCC 8767	–	ON563176	ON621680	ON676524	ON676512	ON568695	Wang et al. 2022
*S. subasiatica*	HKAS144400^T^	PQ492704	PQ492711	PQ492343	PQ499069	PQ499075	PQ499082	Bu et al. 2025
*S. suiyangensis*	SY09821^T^	PV082775	–	PV082892	PV171293	PV171157	PV171211	Chen et al. 2025
*S. suiyangensis*	SY09822	PV082776	–	PV082893	PV171294	PV171158	PV171212	Chen et al. 2025
*S. tiankengensis*	KY 11741^T^	ON502840	–	ON502838	ON525437	–	ON525436	Chen et al. 2022
*S. tiankengensis*	KY 11742	ON502849	–	ON502841	ON525439	–	ON525438	Chen et al. 2022
*S. tongrenensis*	TR05131 ^T^	PV082777	–	PV082894	PV171295	PV171159	PV171213	Chen et al. 2025
*S. tongrenensis*	TR05132	PV082778	–	PV082895	PV171296	PV171160	PV171214	Chen et al. 2025
*S. torquatistipitata*	HKAS144411^T^	PQ492706	PQ492713	PQ492345	PQ499071	PQ499077	PQ499084	Bu et al. 2025
*S. torquatistipitata*	HKAS144402	PQ492705	PQ492712	PQ492344	PQ499070	PQ499076	PQ499083	Bu et al. 2025
*S. tortricidae*	YFCC 6142	–	MN576752	MN576808	MN576978	MN576868	MN576922	Wang et al. 2020
*S. tortricidae*	YFCC 6131^T^	–	MN576750	MN576806	MN576976	MN576866	MN576920	Wang et al. 2020
*S. vallis*	DY07241^T^	OR263159	–	OR263306	OR282778	OR282772	OR282774	Chen et al. 2023
*S. winandae*	MY12469.01^T^	OM491228	–	OM491231	OM687896	OM687901	OM687899	Crous et al. 2023
*S. wudangensis*	WD04121^T^	PV082779	–	PV082896	PV171297	–	–	Chen et al. 2025
*S. wudangensis*	WD04122	PV082780	–	PV082897	PV171298	–	–	Chen et al. 2025
*S. yuanzuiensis*	NTUPPMCC 20-064^T^	MT974206	–	MT974359	–	MW200225	MW200234	Chuang et al. 2024
*S. yuanzuiensis*	NTUPPMCC 20-065	MT974207	–	MT974360	MW200217	MW200226	MW200235	Chuang et al. 2024
*S. yunnanensis*	YFCC 1527^T^	–	MN576756	MN576812	MN576982	MN576872	MN576926	Wang et al. 2020
*S. yunnanensis*	YFCC 1824	–	MN576757	MN576813	MN576983	MN576873	MN576927	Wang et al. 2020
*S. zongqii*	DY45251^T^	PV082781	–	PV082898	PV171299	PV171161	PV171215	Chen et al. 2025
*S. zongqii*	DY45252	PV082782	–	PV082899	PV171300	PV171162	PV171216	Chen et al. 2025
*Tolypocladium ophioglossoides*	NBRC 106330	JN943321	JN941734	JN941407	AB968603	JN992468	AB968564	Ban et al. 2015
*T. ophioglossoides*	NBRC 100998	JN943319	JN941735	JN941406	AB968602	JN992469	AB968563	Ban et al. 2015

Boldface: data generated in this study, ^T^ ex-type material, – means no data.

## ﻿Results

### ﻿Phylogenetic analyses

**Figure 1.**
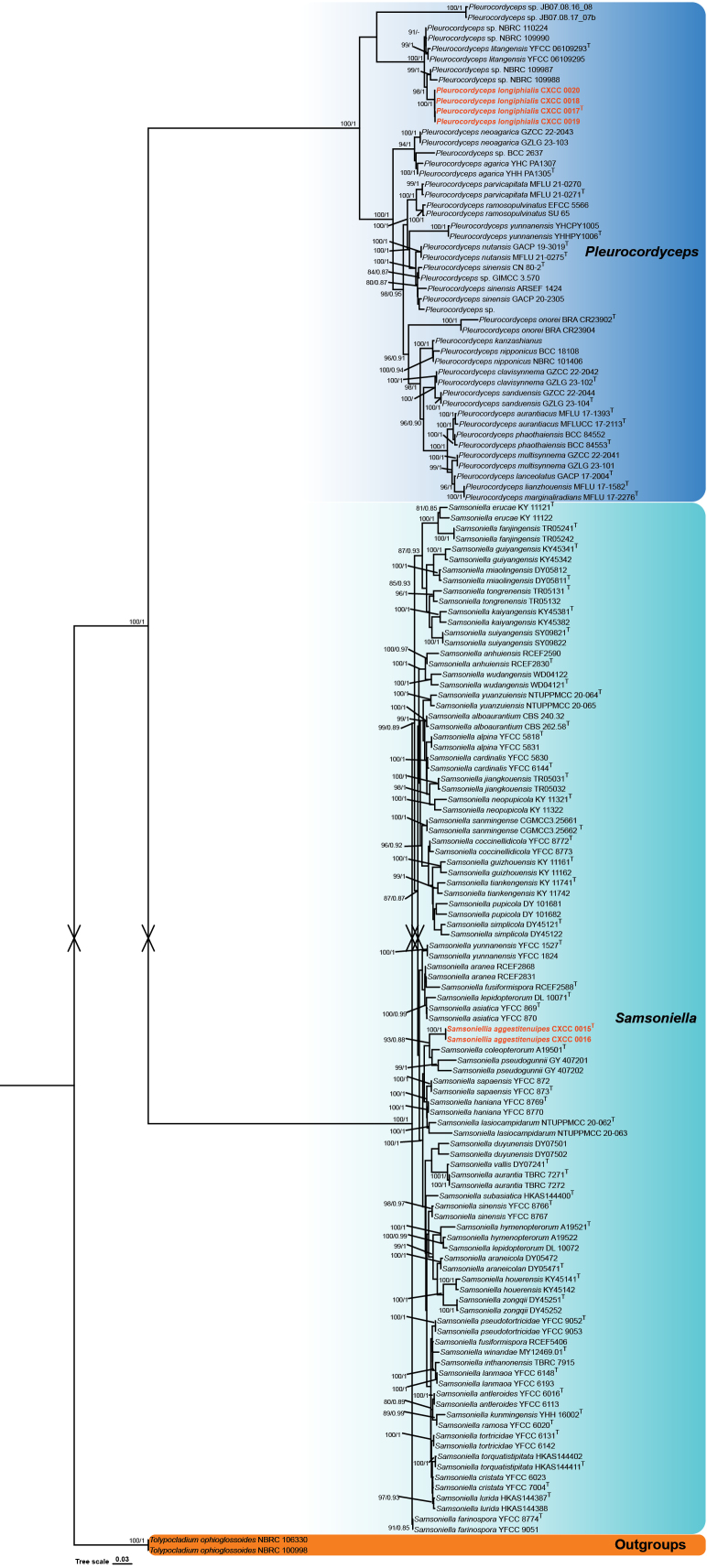
Molecular phylogenetic analyses using the ML and BI based on combined ITS, nr SSU, nr LSU, *tef1-α*, *rpb1*, and *rpb2* sequence data. Two strains of *Tolypocladium ophioglossoides* (NBRC 106330 and NBRC 100998) were used as outgroup taxa. Statistical support values (BS ≥ 80% and PP ≥ 0.80) are shown at the nodes for ML bootstrap support (BS) and BI posterior probabilities (PP). Isolates in red type are those analyzed in this study. The scale bar represents the expected number of changes per site.

The phylogenetic analysis of *Pleurocordyceps* and *Samsoniella* species was performed using a data matrix comprising sequences from 149 samples (Table 2). Two strains of *Tolypocladium ophioglossoides* (NBRC 106330 and NBRC 100998) were used as the outgroups. The final aligned dataset had a total length of 6,034 bp (including gaps), with the following partitions: ITS 768 bp, nr SSU 1,449 bp, nr LSU 913 bp, *tef1-α* 1,022 bp, *rpb1* 732 bp, and *rpb2* 1,150 bp. Both BI and ML analyses generated trees with congruent topologies, in which *Pleurocordyceps* and *Samsoniella* formed distinct and well-supported clades (Fig. 1). The overall tree topologies were consistent with those reported in previous studies. The two newly discovered species were placed within well-supported clades in their respective genera: *P. longiphialis* clustered with *P. litangensis* and an undescribed *Pleurocordyceps* sp. (strains NBRC 109987, 109988, 109990, and 110224), while *S. aggestitenuipes* grouped with *S. coleopterorum* and *S. pseudogunnii*. Each of the new species formed a separate, distinct branch within its respective clade, clearly distinguishing them from their closely related species (Fig. 1).

### ﻿Morphological features

**Figure 2.**
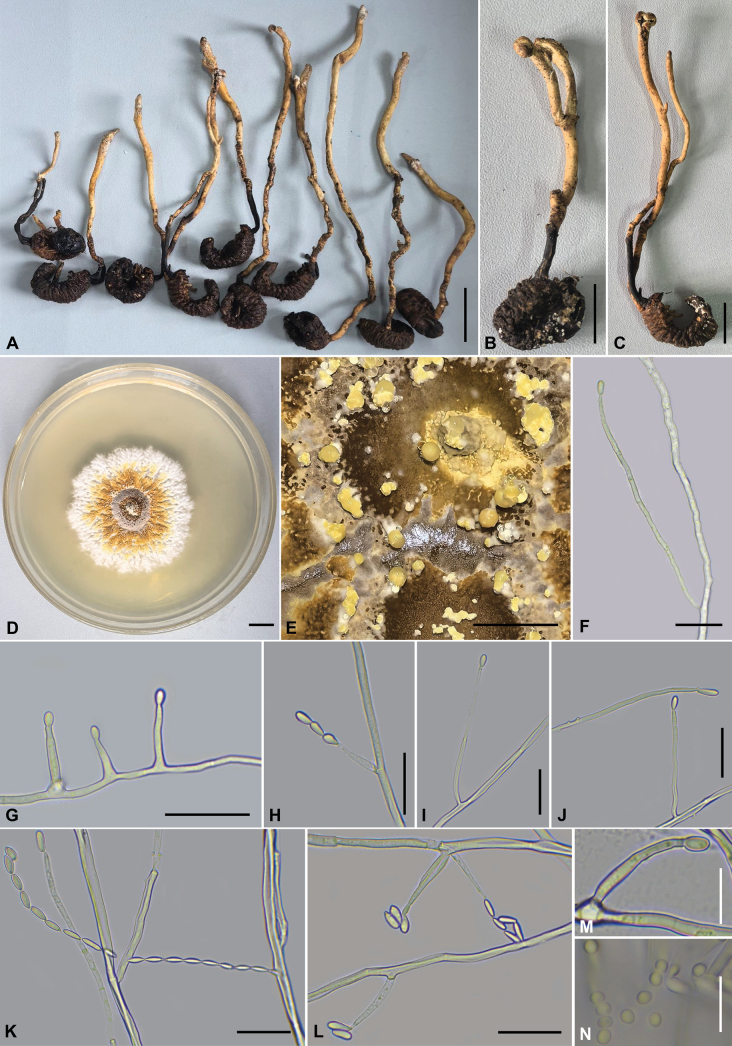
Morphology of *Pleurocordyceps longiphialis* (holotype CXAC 0017; ex-type culture CXCC 0017). **A–C.** Overview of *Pleurocordyceps*; **D.** Colonies on PDA after one month; **E.** Synnemata on the culture; **F, G, I, J, M.** α-phialides; **H, L.** β-phialides and β-conidia; **K.** β-conidia; **N.** α-conidia. Scale bars: 20 mm (**A**); 10 mm (**B, C**); 10 mm (**D**); 5 mm (**E**); 20 µm (**F–L**); 10 µm (**M, N**).

**Figure 3.**
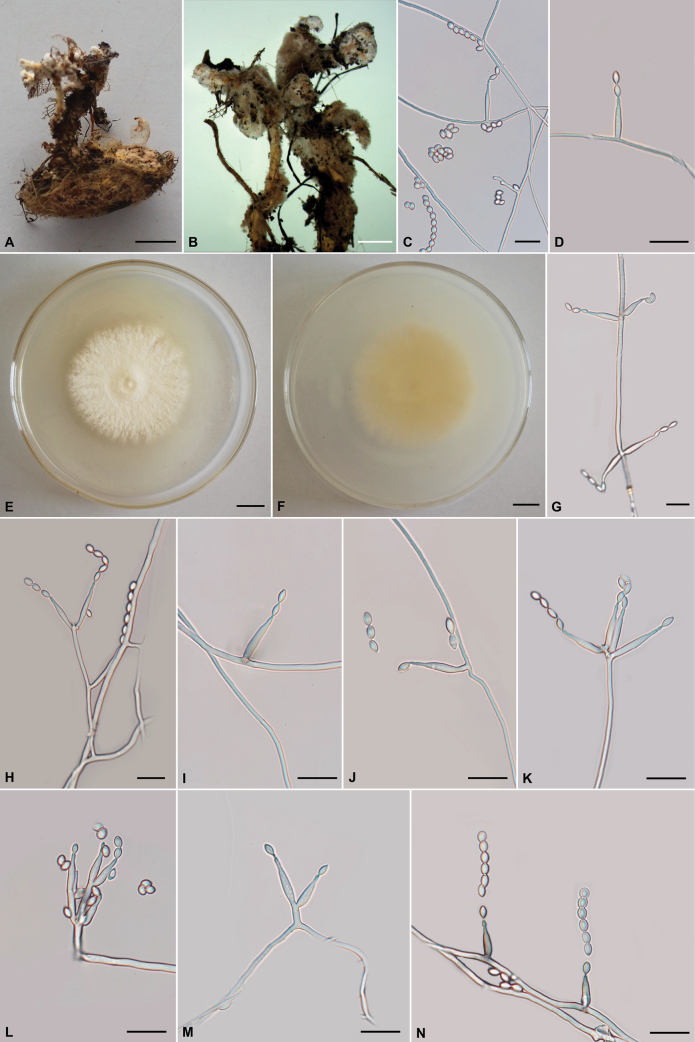
Morphological features of *Samsoniella aggestitenuipes* (holotype CXAC 0015; ex-type culture CXCC 0015). **A.** Synnemata growing on a lepidopteran pupa; **B.** Conidia aggregating at the apex of synnemata; **C, D, G–N.** Phialides with conidia on PDA; **E, F.** Colonies on PDA after 21 days (**E.** Obverse; **F.** Reverse). Scale bars: 10 mm (**A, E, F**); 3 mm (**B**); 10 µm (**C, D, G–N**).

The morphological characteristics and photomicrographs of the two newly described species, *Pleurocordyceps longiphialis* (Polycephalomycetaceae) and *Samsoniella aggestitenuipes* (Cordycipitaceae), are presented in Figs 2, 3, with comprehensive descriptions provided in the Taxonomy section.

### ﻿Taxonomy

Two new species are described in this study.

Table 3 lists all hosts, substrates, and geographical locations of *Pleurocordyceps* species.

**Table 3.** *Pleurocordyceps*, hosts, substrates, and geographical location.

Species	Host/Substrate	Countries found	References
*P. agarica*	*Ophiocordyceps* sp.	China	Wang et al. 2015a
*P. aurantiaca*	Coleoptera larvae or *Ophiocordyceps barnesii*, *Ophiocordyceps neouguetii*	Thailand	Xiao et al. 2018
*P. clavisynnema*	*Ophiocordyceps neogryllotalpae*	China	Xiao et al. 2024
*P. fusiformispora*	*Ophiocordyceps* sp.	China	Liu et al. 2024a
*P. heilongtanensis*	*Ophiocordyceps* sp.	China	Xiao et al. 2023
*P. lanceolata*	Lepidoptera larvae	China	Xiao et al. 2023
*P. lianzhouensis*	Lepidoptera larva or *Ophiocordyceps crinalis*	China	Wang et al. 2014
*P. litangensis*	*Ophiocordyceps sinensis*	China	Liu et al. 2024b
***P. longiphialis***	**Scarabaeoidea larvae**	**China**	**This study**
*P. marginaliradians*	Cossidae larva	Thailand	Xiao et al. 2018
*P. multisynnema*	*Paraisaria* sp.	China	Xiao et al. 2024
*P. neoagarica*	*Ophiocordyceps neogryllotalpae*	China	Xiao et al. 2024
*P. nipponica*	*Graptopsaltria nigrofiscata* (Hemiptera)	Japan	Kobayasi 1939
*P. nutantis*	*Ophiocordyceps nutans*, Hemiptera larvae	China	Xiao et al. 2023
*P. onorei*	Lepidoptera larva	Ecuado	Crous et al. 2017b
*P. ophiocordycipiticola*	*Ophiocordyceps cylindrospora*	Thailand	Wijayawardene et al. 2022
*P. parvicapitata*	*Perennicordyceps elaphomyceticola*, *Elaphomyces* sp.	China	Xiao et al. 2023; Wang et al. 2024b
*P. phaothaiensis*	Coleoptera larva	Thailand	Crous et al. 2017a
*P. puerensis*	Coleoptera larva	China	Cao et al. 2025
*P. ramosopulvinata*	Cicadidae	Japan	Kobayasi and Shimizu 1983
*P. sanduensis*	*Ophiocordyceps neogryllotalpae*	China	Xiao et al. 2024
*P. sinensis*	Lepidoptera larvae, *Ophiocordyceps crinalis*, *Ophiocordyceps barnesii*, *Ophiocordyceps neoacicularis*, *Ophiocordyceps sinensis*	China	Chen et al. 1984; Wang et al. 2012
*P. vitellina*	*Ophiocordyceps nigrella*	China	Xiao et al. 2023
*P. yunnanensis*	Hemiptera adults or *Ophiocordyceps nutans*	China	Wang et al. 2015b; Wang et al. 2024b

#### ﻿ Pleurocordyceps longiphialis

Taxon classificationFungiHypocrealesOphiocordycipitaceae

Q.Y. Dong & C.D. Xu sp. nov.

0F58F123-6EF7-5709-8EF6-982EDFD2034D

860988

Fig. 2

##### Etymology.

The specific epithet *longiphialis* refers to the characteristically elongated phialides, which distinguish this species from its closely related congeners.

##### Holotype.

China, • Guizhou Province, Qiandongnan Miao and Dong Autonomous Prefecture, Shibing County, Chengguan Town, on the larva of Scarabaeoidea, 25 March 2025, Quanying Dong (holotype CXAC 0017; ex-holotype living culture, CXCC 0017).

##### Stromata morph.

Host a Scarabaeoid larva, 47–32.5 × 3–5 mm, swollen, cylindrical, black. ***Stromata*** arise from the head and thoracic region of the host, solitary or paired, simple or branched, clavate, yellow to brown, 47–142.5 × 1.5–3.5 mm. In some specimens, the stromatal apex expands into a fist to globose-shaped head, occasionally fissuring into two or three lobes; in others, it divides into two segments, one longer and thicker, the other shorter and narrower. Certain stromata bear a black stipe, 6.5–17 mm long. Sterile.

##### Culture characters.

Colony on PDA attaining a diameter of 42–44 mm after one month at 25 °C. Colony cottony, fluffy, with high mycelial density, central pale brownish-white umbonate, surrounded by successive concentric rings: an inner dark brown band, a broader light to dark brown zone, a distinct yellow ring, and an off-white periphery with a yellowish tint; reverse dark brown center and creamy-white margin. ***Hyphae*** smooth walled, branched, septate, hyaline, 1–2.5 µm wide. ***Synnemata*** emerging after two months, clavate or with a mucronate apex, solitary, unbranched, and 2–5 mm long, 0.5–2.5 mm wide, yellowish to yellow, emerging on the middle part of the synnemata or on the top, with conidial masses on the surface (Fig. 2E). Terminal portion of a synnemata covered by a viscous mass. Cultures readily produced phialides and conidia on potato dextrose agar after 14 days at 25 °C. ***Conidiophores*** are cylindrical, hyaline, smooth-walled, simple to verticilate form 1–3. ***Phialides*** have two types, α-phialides solitary, cylindrical or lanceolate, tapering gradually from base to apex, 9.5–101 × 2–3.5 µm, 2–3.5 µm wide (base), 1–2 µm wide (apex). β-phialides terminal on solitary on hyphae; lageniform or subulate, tapering abruptly from the base to the apex, 7.5–15.5 × 1–2.5 µm, 1–2.5 µm wide (base), 0.5–1.5 µm wide (apex). α-conidia one-celled, hyaline, smooth, subglobose, ovoid to ellipsoidal, 2–3 × 2–3 µm. β-conidia one-celled, hyaline, smooth, fusiform, oblong-elliptical to ellipsoidal, 4.5–8.5 × 1.5–3.5 µm, solitary or aggregated in long chains.

##### Host.

Scarabaeoidea larva

##### Known distribution.

Guizhou Province, China.

##### Additional specimens examined.

China, • Guizhou Province, Qiandongnan Miao and Dong Autonomous Prefecture, Shibing County, Chengguan Town, on the larva of Scarabaeoidea, 25 March 2025, Quanying Dong (paratype CXAC 0018; ex-paratype living culture, CXCC 0018). China, • Guizhou Province, Qiandongnan Miao and Dong Autonomous Prefecture, Shibing County, Baiduo Township, isolated from the larva of Scarabaeoidea, 1 April 2025, Quanying Dong (paratype CXAC 0019, CXAC 0020; ex-paratype living culture, CXCC 0019, CXCC 0020).

##### Commentary.

The diagnostic morphology of *Pleurocordyceps longiphialis* conforms to the generic concept of *Pleurocordyceps*. The species produces dimorphic, often verticillately branched phialides, comprising two types: α-phialides, verticillate with a cylindrical to subulate base tapering into a long neck, and β-phialides, solitary, narrow, lageniform to subulate, and tapering abruptly. Conidia are also dimorphic: α-conidia are globose to subglobose or ellipsoidal, while β-conidia are fusiform and occur solitary or in chains. The species can be distinguished by the following combination of morphological characteristics: α-phialides cylindrical to lanceolate, 9.5–101 × 2–3.5 μm; β-phialides lageniform to subulate, 7.5–15.5 × 1–2.5 μm; α-conidia subglobose, ovoid to ellipsoidal, 2–3 × 2–3 μm; and β-conidia fusiform, oblong-elliptical to ellipsoidal, 4.5–8.5 × 1.5–3.5 μm, occurring solitary or aggregated in long chains. Furthermore, this species was isolated from a Scarabaeoidea larva.

Phylogenetically, *Pleurocordyceps longiphialis* is strongly supported (BS = 100%, PP = 1) and clusters with *P. litangensis* (strains YFCC 06109293 and YFCC 06109295) and an undescribed *Pleurocordyceps* sp. (strains NBRC 109987, 109988, 109990, and 110224). However, it forms a distinct clade separate from these taxa (Fig. 1). Morphologically, *P. longiphialis* differs from related species in several aspects. *P. litangensis*, described from Litang County, Sichuan Province, China, possesses aculeate and relatively shorter α-phialides (11.2–12.8 μm vs. 9.5–101 μm in *P. longiphialis*), longer β-phialides (9.9–27.8 μm vs. 7.5–15.5 μm), larger α-conidia (3.2–6.1 × 1.8–3.9 μm vs. 2–3 × 2–3 μm), and smaller β-conidia (3.5–6.1 × 1.4–2.5 μm vs. 4.5–8.5 × 1.5–3.5 μm). Ecologically, *P. litangensis* is reported from the host *Ophiocordyceps sinensis* (Ophiocordycipitaceae), whereas *P. longiphialis* is associated with a Scarabaeoidea larva (Liu et al. 2024b).

Table 4 lists all hosts, substrates, and geographical locations of *Samsoniella*.

**Table 4.** Hosts, substrates, and geographic distribution of *Samsoniella*.

Species	Host/Substrate	Countries found	References
***S. aggestitenuipes***	**Lepidoptera pupa**	**China**	**This study**
*S. alboaurantia*	Soil, Lepidoptera pupa	England	Smith 1957
*S. alpina*	*Hepialus baimaensis* larvae	China	Wang et al. 2020
*S. anhuiensis*	Spider	China	Wang et al. 2024a
*S. antleroides*	Noctuidae larvae	China	Wang et al. 2020
*S. aranea*	Spider	China	Wang et al. 2024a
*S. araneicola*	Spider	China	Chen et al. 2025
*S. asiatica*	Lepidoptera pupa	China, Laos, Vietnam	Wang et al. 2023
*S. aurantia*	Lepidoptera larvae	China, Thailand, Vietnam	Mongkolsamrit et al. 2018; Wang et al. 2023
*S. cardinalis*	Limacodidae pupae	China	Wang et al. 2020
*S. coccinellidicola*	Coccinellidae (Coleoptera)	China	Wang et al. 2022
*S. coleopterorum*	Snout beetle (Coleoptera)	China	Chen et al. 2020
*S. cristata*	Saturniida pupae (Lepidoptera)	China	Wang et al. 2020
*S. doupengshanensis*	Lepidoptera pupa	China	Zeng et al. 2025
*S. duyunensis*	Formicidae, Lepidoptera pupa	China	Chen et al. 2023
*S. erucae*	Lepidoptera (Caterpillar)	China	Chen et al. 2022
*S. fanjingensis*	Lepidoptera pupa	China	Chen et al. 2025
*S. farinospora*	Spider, Lepidoptera: Hepialus	Vietnam	Wang et al. 2022
*S. formicae*	Ant (Formicidae)	China	Chen et al. 2022
*S. fusiformispora*	Spider	China	Wang et al. 2024a
*S. guiyangensis*	Lepidoptera pupa	China	Chen et al. 2025
*S. guizhouensis*	Lepidoptera pupa	China	Chen et al. 2022
*S. haniana*	Lepidoptera pupa	China	Wang et al. 2022
*S. hepiali*	Lepidoptera pupa, larvae	China, Vietnam, Argentina	Wang et al. 2023
*S. houerensis*	Lepidoptera pupa	China	Chen et al. 2025
*S. hymenopterorum*	Vespidae	China	Chen et al. 2020
*S. inthanonensis*	Lepidopterous larvae	Thailand, China	Mongkolsamrit et al. 2018
*S. jiangkouensis*	Lepidoptera pupa	China	Chen et al. 2025
*S. kaiyangensis*	Lepidoptera pupa	China	Chen et al. 2025
*S. kunmingensis*	Lepidoptera pupa	China	Wang et al. 2020
*S. lanmaoa*	Lepidoptera pupa	China	Wang et al. 2020
*S. lasiocampidarum*	Lasiocampidae (Lepidoptera)	China	Chuang et al. 2024
*S. lepidopterorum*	Lepidoptera pupa	China	Chen et al. 2020
*S. lurida*	Lepidoptera cocoon, larva	China	Bu et al. 2025
*S. miaolingensis*	Lepidoptera pupa	China	Chen et al. 2025
*S. neopupicola*	Lepidoptera pupa	China	Chen et al. 2022
*S. pseudogunnii*	Lepidoptera larvae	China	Chen et al. 2021
*S. pseudotortricidae*	Lepidoptera pupa	China	Wang et al. 2022
*S. pupicola*	Lepidoptera pupa	China	Chen et al. 2021
*S. ramosa*	Limacodidae pupa	China	Wang et al. 2020
*S. sanmingensis*	Lepidoptera larvae	China	Pu et al. 2025
*S. sapaensis*	Lepidoptera larvae	Vietnam	Wang et al. 2023
*S. scoliopterygis*	*Scoliopteryx libatrix*	UK, Ireland	Evans et al. 2025
*S. simplicola*	Lepidoptera pupa	China	Chen et al. 2025
*S. sinensis*	Lepidoptera larvae, Dermaptera	China	Wang et al. 2022
*S. subasiatica*	Lepidoptera pupa	China	Bu et al. 2025
*S. suiyangensis*	Cicadellidae	China	Chen et al. 2025
*S. tiankengensis*	Lepidoptera pupa	China	Chen et al. 2022
*S. tongrenensis*	Lepidoptera pupa	China	Chen et al. 2025
*S. torquatistipitata*	Ant	China	Bu et al. 2025
*S. tortricidae*	Tortricidae pupa (Lepidoptera)	China	Wang et al. 2020
*S. vallis*	Lepidoptera pupa	China	Chen et al. 2023
*S. winandae*	Lepidoptera pupa	Thailand	Crous et al. 2023
*S. wudangensis*	Lepidoptera larvae	China	Chen et al. 2025
*S. yuanzuiensis*	Lepidoptera pupa	China	Chuang et al. 2024
*S. yunnanensis*	Limacodidae pupa, *Cordyceps* sp., *Cordyceps cicadae*	China	Wang et al. 2020
*S. zongqii*	Lepidoptera larvae	China	Chen et al. 2025

#### ﻿ Samsoniella aggestitenuipes

Taxon classificationFungiHypocrealesCordycipitaceae

Q.Y. Dong & S.Y. Gao sp. nov.

9A1037E9-FFB2-5DCD-BEDA-6980B52D124D

860989

Fig. 3

##### Etymology.

The epithet *aggestitenuipes* combines *aggesti*-, from Latin *aggestus*, referring to the conidial accumulation, with *tenuipes*, from *Cordyceps tenuipes*, reflecting a morphology similar to *C. tenuipes* but distinguished by synnematal branches that bear massive conidial aggregates.

##### Holotype.

China, • Sichuan Province, Chengdu City, Qionglai City, Tiantaishan County, isolated from the pupa of Lepidoptera, 21 August 2024, Quanying Dong (holotype CXAC 0015; ex-holotype living culture, CXCC 0015).

##### Sexual morph.

Undetermined.

##### Culture characters.

Conidial arrangement *Isaria*-like. Synnemata two or several, on the pupae of Lepidoptera buried in soil, white, up to 1.8–2.7 cm long. Stipes cylindrical, 0.6 mm wide, producing a mass of conidia at the branches of synnemata, powdery.

Colonies on PDA attaining a diameter of 49–52 mm in 21 days at 25 °C, white to cream-colored, soft cottony aerial mycelium, reverse pale yellow. Hyphae smooth-walled, branched, septate, hyaline, 1–2 µm wide. Synnemata arising from the middle body of pupae were irregularly branched, 1.8–2.7 cm long, 0.3–0.6 mm wide; cylindrical or clavate stipes with powdery white heads. Cultures readily produced phialides and conidia after 1 week on potato dextrose agar at 25 °C showing a granular appearance due to profuse conidiation. Conidiophores cylindrical or clavate, hyaline, smooth-walled, simple to verticilate form 1–3, 5–10.5 × 2–2.5 µm, 1–2 µm wide (base), 2–2.5 µm wide (apex). Phialides from aerial mycelium straight to slightly flexuose, solitary or in whorls of two to four on each branch, cylindrical to flask-shaped, usually with a slightly swollen basal part, 6–28 × 1–2.5 µm, tapering gradually or abruptly from 1–2 µm (base) to 0.5–1.5 µm (apex). Conidia hyaline, subglobose, ovoid, or fusiform, smooth, one-celled, 2–4 ×1.5–2.5 µm, usually in chains.

##### Other material examined.

China, • Sichuan Province, Chengdu City, Qionglai City, Tiantaishan County, on the pupa of Lepidoptera, 21 August 2024, Quanying Dong (paratype CXAC 0016; ex-paratype living culture CXCC 0016).

##### Host.

Lepidoptera pupa.

##### Known distribution.

Sichuan Province, China.

##### Commentary.

*Samsoniella aggestitenuipes* displays typical genus-level characteristics such as solitary or whorled phialides and conidia varying from subglobose to ovoid or ellipsoid. It is distinguished by the following unique combination of morphological characteristics: phialides solitary or in whorls of 2–4, cylindrical, 6–28 × 1–2.5 µm; conidia mostly subglobose, ovoid, or fusiform, 2–4 × 1.5–2.5 µm, usually aggregated in chains. Furthermore, the species was isolated from a Lepidoptera pupa. Phylogenetically, *S. aggestitenuipes* is strongly supported (BS = 93%, PP = 0.88) and clusters with *S. coleopterorum* and *S. pseudogunnii*, but it is distinguished from these two species by forming a separate clade within this group (Fig. 1). Morphologically, these two species differ from *S. aggestitenuipes* in the following ways. *Samsoniella coleopterorum*, a species described from China, has aculeate and relatively shorter phialides measuring 5.4–9.7 × 1.2–1.8 μm and smaller conidia, 1.7–2.5 × 1.2–1.8 μm vs. 2–4 × 1.5–2.5 µm (Chen et al. 2020). *Samsoniella pseudogunnii*, a species described from Guiyang, Guizhou Province, and also similar to *S. aggestitenuipes* in appearance, has relatively shorter phialides (6.8–11 × 2.2–2.4 µm vs. 6–28 × 1–2.5 µm) and relatively smaller conidia (2.8–3.2 × 1.7–2.1 µm vs. 2–4 × 1.5–2.5 µm) (Chen et al. 2021).

## ﻿Discussion

Employing an integrative taxonomic framework of molecular phylogenetics and morphology, we describe two new entomopathogenic fungi from China: *Pleurocordyceps longiphialis* and *Samsoniella aggestitenuipes*. This discovery deepens our understanding of hypocrealean diversity while providing fresh perspectives on host specificity and biogeography. The strong phylogenetic distinction between the species thereby affirms the value of a multilocus methodology for defining species boundaries in these morphologically complex groups.

### ﻿Host diversity and economic value

Both *Pleurocordyceps* and *Samsoniella* exhibit broad host ranges and ecological versatility. *Pleurocordyceps* infects insects across multiple orders (Coleoptera, Hemiptera, and Lepidoptera) and also parasitizes fungi such as *Elaphomyces*, *Ophiocordyceps*, *Paraisaria*, and *Perennicordyceps* (Table 3). This dual ecological strategy highlights remarkable adaptability. The discovery of *P. longiphialis* on a Scarabaeoidea larva extends the genus’ host range to this coleopteran group, revealing a previously unreported niche. Notably, its closest relative, *P. litangensis*, parasitizes *Ophiocordyceps sinensis* (Liu et al. 2024b). The observed variation in host use among related *Pleurocordyceps* species suggests notable ecological plasticity. This pattern could reflect a potential link between host switching from fungi to insects and diversification within the genus, a hypothesis that requires further phylogenetic and genomic investigation.

*Samsoniella* shows similar host plasticity, infecting Lepidoptera, Coleoptera, Hymenoptera, and Arachnida (Table 4). Although *S. aggestitenuipes* was isolated from a lepidopteran pupa, a common host among related species, its phylogenetic distinction and morphological traits, such as elongated phialides and unique synnemata, indicate niche specialization. The coexistence of generalist and specialist lineages underscores evolutionary flexibility in host–pathogen interactions within these fungi.

Beyond their ecological significance, the genera *Pleurocordyceps* and *Ophiocordyceps* are of considerable economic importance. *Pleurocordyceps* species produce valuable bioactive metabolites such as antioxidants and antimicrobials from *Pl. nipponica* and anti-inflammatory agents from *P. phaothaiensis*, highlighting their potential for pharmaceutical and industrial use (Sangdee et al. 2017; Somsila et al. 2018; Sonyot et al. 2020).

*Ophiocordyceps sinensis* is a renowned medicinal fungus in traditional Chinese medicine. However, its natural scarcity and high cost limit sustainable utilization. Recent discoveries of novel sterols and immunomodulatory polysaccharides in *O. sinensis* continue to unveil its complex bioactive foundation (Yao et al. 2024; Qian et al. 2025; Wang et al. 2025). A viable alternative, *Samsoniella hepiali*, was isolated in 1982 from *O. sinensis* collected in Yunnan Province. It mirrors the chemical and pharmacological profile of natural *O. sinensis*, exhibiting analgesic, anti-aging, immunomodulatory, hypoglycemic, nephroprotective, anti-inflammatory, and antidepressant activities. Approved as a health food ingredient in 2001, it is used in the commercial product “Jinshuibao Capsule,” with current annual sales of related products reaching 3 billion CNY (Dai et al. 1989; Wang et al. 2017; Ge et al. 2018; Zhao et al. 2020; Li et al. 2022). Furthermore, *S. hepiali* serves as a key component in over 260 health products globally, contributing to an estimated yearly market value of CNY 10 billion (Wang et al. 2020). Continued exploration of these genera therefore offers significant opportunities for discovering new bioactive compounds and expanding their economic applications.

### ﻿Geographic distribution and endemism

Both genera exhibit wide geographic distributions consistent with their broad host ranges. *Pleurocordyceps* occurs in Asia (China, Japan, and Thailand) and South America (Ecuador) (Table 3), while *Samsoniella* is found across East Asia (China, Laos, Thailand, and Vietnam), Europe (UK and Ireland), and South America (Argentina) (Table 4). A striking concentration of species discoveries, particularly of *Samsoniella*, centers in southwestern China, most notably Yunnan Province. This biodiversity hotspot appears to act as a diversification center for these fungi, likely promoted by complex topography, diverse ecosystems, and abundant insect hosts.

The recent discoveries of *P. longiphialis* in Guizhou and *S. aggestitenuipes* in Sichuan, both bordering Yunnan, underscore the incomplete documentation of regional fungal diversity, indicating its likely extension beyond currently known ranges. These biogeographic patterns, characterized by widely distributed genera with multiple presumed endemic species, reflect a complex evolutionary history involving both dispersal and isolation. However, sampling bias across regions hinders clear differentiation between true endemism and sampling artifacts.

This study demonstrates the efficacy of integrative taxonomy in revealing cryptic fungal diversity. The newly described *P. longiphialis* and *S. aggestitenuipes* offer novel insights into the evolution of Hypocreales, illustrating how host shifts and geographic isolation serve as key drivers of speciation. The extensive host ranges and distinctive biogeographic distributions of genera such as *Pleurocordyceps* and *Samsoniella* further reflect their high ecological adaptability. Finally, the biosynthetic potential of these and related taxa warrants further investigation, as they may represent promising sources of novel bioactive compounds for agricultural and pharmaceutical applications.

## Supplementary Material

XML Treatment for Pleurocordyceps longiphialis

XML Treatment for Samsoniella aggestitenuipes

## ﻿Additional information

### Use of AI

No use of AI was reported.

### Data availability

All of the data that support the findings of this study are available in the main text.
